# Be Considerate

**Published:** 1878-06-20

**Authors:** 


					﻿EDITOElAL AND MISCELLANEOUS.
BE CONSIDERATE.
Friends, those of you who have not paid your
subscription are requested to do so at once. Con-
sider how much easier for you to raise $2 one
time in the year, than for us to raise a hundred
every month.
				

